# Healing Pathways: A Program for Women with Physical Disabilities and Depression

**DOI:** 10.1155/2013/649875

**Published:** 2013-05-02

**Authors:** Dena Hassouneh, Thuan Nguyen, Zunqiu Chen, Elizabeth McNeff

**Affiliations:** ^1^School of Nursing, Oregon Health & Science University, 3455 SW US Veteran's Hospital Road, Portland, OR 97239, USA; ^2^School of Public Health and Preventive Medicine, Oregon Health & Science University, 3181 SW Sam Jackson Park Road, CB 669, USA; ^3^Regional Research Institute, Portland State University, 600 SW 4th Avenue, Suite 900, Portland, OR 97201, USA

## Abstract

*Objective*. The objective of this study was to test the efficacy of the Healing Pathways (HP) program in reducing clinically significantly depressive symptoms in women with physical disabilities (WPD). Healing Pathways is a peer-implemented group mental health treatment program targeting WPD who have clinically significant cooccurring depressive symptoms. *Participants*. Eighty women were randomized in this trial. *Design*. This study used a community-based participatory intervention research design. Using community-based recruiting methods, participants were recruited from Centers for Independent Living, local disability service organizations, via Craig's list as well as other community locations such as grocery stores and bus stops. Women participated in the HP program for 14 weeks. *Results*. The primary outcome variable for this study was reduction in depressive symptoms as measured by the Center for Epidemiologic Depression Scale (CES-D). We found that there was a significant interaction effect of treatment by time on depression scores, *F*(3,210) = 9.51, *P* < 0.0001, partial *η* ^2^ = 0.101. Investigation of the predicted mean profile over time in the intervention group demonstrated that depression scores decreased greatly from baseline to the first posttest and remained stable in the two followups, whereas there was a little change in the mean profile over time in the control group. *Conclusion*. The HP program has demonstrated initial efficacy in reducing depressive symptoms in women with physical disabilities.

## 1. Introduction

 Women are at least twice as likely as men to experience a major depressive episode once in their lifetimes and approximately 70% of the prescriptions written for antidepressants are given to women [1]. The reasons for gender disparities in depression rates include the influence of sex hormones, and the incidence of serious adverse life events such as childhood and adult sexual abuse and male partner violence [2, 3]. Even in the absence of physical disability, depression significantly impairs women's social and physical functioning [1, 2]. Because many women with physical disabilities (WPD) have poorer health than nonphysically disabled women, they often have fewer reserves to compensate for depressive symptoms [4–6]. Thus, when depression and physical disability cooccur, the effects are usually most severe. 

 Depression negatively affects almost every conceivable outcome for women with physical disabilities (WPD), from physical health and functioning to employment, quality of life, and mortality [7–15]. This is alarming since women with physical disabilities WPD experience clinically significant depressive symptoms at high rates and may face barriers to accessing needed care. In a study of 443 WPD, Hughes and colleagues found that 51% of the sample had scores signifying mild depression or higher on the Beck Depression Inventory (BDI) [4, 16]. Of those women who were depressed, only 44% had been able to access treatment within the past three months [4]. In a second study of 64 women living with spinal cord injury for at least two years, Hughes and colleagues found that 59.1% of women had clinically significant depressive symptoms as measured by the Center for Epidemiologic Studies Depression Scale (CES-D) [7, 17]. In our study of abuse and health in men and women with spinal cord injury or dysfunction, 53% of our sample of 165 women had clinically significant depressive symptoms as measured by the CES-D [18]. 

 Consistent with findings cited above, Hughes and colleagues study of 134 depressed WPD found that more than a third of the women had received no treatment for depression within the past three months [19]. Barriers to accessing mental health care that WPD may face include physical illness, transportation, accessibility, and poverty [19]. Another important barrier is the lack of mental health treatment programs available that address the experience of cooccurring physical disability and depression. Although a small number of programs focus on depression as a primary outcome [20–25], we found only one published paper describing a program that builds on WPD's shared experiences and the strengths of disability communities [20]. 

 Although WPD are a diverse group, there are some common variables of interest that are associated with depressive symptoms. Coping and health behaviors have been widely cited as important constructs for WPD in terms of their overall health and well-being and the prevention and amelioration of secondary conditions like depression [5, 26–29]. Active coping [26], spirituality, interpersonal relations, and stress management in particular have been mentioned [5]. Self-esteem both as a global construct and specific to a woman's body and sexuality have also been identified as important for understanding depressive symptoms. According to Hughes and colleagues, “lowered self-esteem is a common feature of depression” in WPD [30, page 296]. In their study to determine the efficacy of a 6-week self-esteem group intervention for WPD, they found that self-esteem mediated the effect of the intervention on depression [30]. In a study of body and sexual self-esteem, Taleporos and McCabe sampled 1196 participants including 748 persons with physical disabilities and 448 persons without physical disabilities [31]. They found that sexual and body esteem were strong predictors of self-esteem and depression in people with physical disabilities and that this effect was stronger among people with disabilities than in people without disabilities. Loneliness or social participation has also been identified as an important variable related to depression in WPD [32, 33]. In a study of the relevance of depressive symptoms and social support to disability in women with multiple sclerosis (*n* = 118) and fibromyalgia (*n* = 197), Phillips and Stuifbergen found that social support had a large protective effect on depressive symptoms [33]. The authors noted that interventions that promote social connection may particularly be important for WPD. 

 To help address the serious problem of depression in WPD, to expand available treatment options, and to increase access to affordable treatment we developed and tested the Healing Pathways program using a community-based participatory research design (CBPR). Healing Pathways (HP) is a cross-disability peer implemented strengths-based cognitive behavioral group treatment program. To assess the efficacy of the HP program we conducted a study in partnership with three grass roots consumer-run disability service agencies. The purpose of CBPR is to engage researchers and community members as equal partners to create knowledge that is directly applicable and available to communities, especially those who have historically experienced social and economic marginalization [34, 35]. Our academic-community partnership grew out of ten years of community-placed work and one previous CBPR project. Guiding principles of our collaboration included accessibility to all aspects of the projects across physical disability types and collaborative development of interventions that enable community control over future implementation. See Hassouneh et al. [36] for more information about the HP partnership and the CBPR methodology employed. 

## 2. Study Aims

 The aims of the study were to (1) modify an existing group therapy intervention to address the specific needs of WPD who experience depressive symptoms [37] and to (2) conduct an efficacy trial of the modified intervention. We hypothesized that WPD receiving the HP program would demonstrate greater improvement in our primary outcome variable depressive symptoms than WPD in the wait-list control group. We also hypothesized that WPD receiving the HP program would demonstrate greater improvement in our secondary outcome variables, coping, health behavior, global self-esteem, body image and sexual self-esteem, and loneliness, compared to WPD in the wait-list control group. Finally we asked the following research question: what are the relative contributions of changes in coping, health behavior, global self-esteem, body image and sexual self-esteem, and loneliness to changes in depressive symptoms? 

## 3. Method

### 3.1. Participants

We screened a total of 101 women and enrolled 89 WPD of age 19–81. Nine women were ineligible and three declined to enroll. Of the 89 WPD enrolled we randomized 80; 44 were assigned to the intervention group and 36 to the wait-list control group. Nine women dropped out prior to the time when randomization occurred due to reasons of illness and moving away. We assessed depressive symptom scores during the initial screening interview using the CES-D. Inclusion criteria for study enrollment included (1) self-reported physical disability requiring some form of disability accommodation and support; and (2) a CES-D score of 16 or higher at the time of enrollment. Exclusion criteria included (1) significant self-reported intellectual disability and/or receipt of developmental disability services; (2) active suicidality; (3) current receipt of other psychotherapy services; and (4) current psychosis. Significant self-reported intellectual disability was ascertained by asking the following two questions: (1) do you have a guardian? and (2) are you receiving development disability services for a cognitive disability? These criteria allowed for inclusion of a wide variety of WPD often with multiple cooccurring conditions. All participants enrolled in the study who completed baseline data were included in the analysis. 

 The demographic and disability-related characteristics of the participants are presented in Table 1. As presented in the table the majority of participants were European-American (75.95%), employed (73.42%), and single (70.51%) with a household income of less than $20,000 per year (81.01%). With regard to disability 64.63% reported having arthritis or other musculoskeletal disorders, 29.27% reported having a neurological disorder, and 43.9% reported having some other kind of condition. The majority of participants rated their disabilities as moderate (40.51%) or severe (56.76%). Ninety percent of participants reported receiving assistance from others to help them with activities they would normally be able to do themselves if they did not have a disability. 

### 3.2. Description of the HP Program

The HP program is a manualized strengths-based cognitive behavioral group therapy program designed to be implemented by two peers. Program materials include a women's workbook and a facilitator manual. The final version of the program includes 14 sessions each of 2.5 hours in length. The PI assumed primary responsibility for development of the HP program in collaboration with WPD from Oregon disability communities over the course of two CBPR projects [36]. Development of the HP program was informed by a complete literature review, a series of focus groups, input from an advisory board, and written contributions from WPD community members. The HP program promotes self-empowerment through skill building in four core areas (1) identifying and addressing thoughts and beliefs; (2) identifying and managing emotions; (3) improving interpersonal skills; and (4) developing and strengthening coping and problem-solving skills. The principles underlying cognitive behavioral therapy and strengths-based approaches to promoting healing and personal growth are woven throughout each of these areas and the entire program. Healing Pathways session topics include: (1) introduction to the program and overview of depression in WPD; (2) strengths and goals; (3) mental habits; (4) understanding and managing emotions; (5) sense of self (includes optional content on body image and sexuality); (6) personal identity and role models; (7) violence and abuse; (8) relationships, social support, and physical disability; (9) developing communication skills; (10) stress and coping; (11) wellness; and (12) moving forward. 

### 3.3. Procedures

#### 3.3.1. Selection of Sites

There were two urban and two rural partners in this project. Our originally urban HP partnership evolved primarily from preexisting partnerships that had developed over time through work on a previously completed community-based participatory research project as well as other community-placed research projects. We added rural partners to broaden the impact of our findings. Characteristics sought among partners were commitment to Independent Living Philosophy, a track record of reliability, and commitment to the goals of the project. Community buy-in was also assessed as part of the selection process through a series of meetings with local leaders. Existing team leaders met to discuss potential new partners and selection decisions were made by consensus. 

#### 3.3.2. Team Training

We provided a 10-day face-to-face training that included all components necessary for completing the project. Team members attended only those components of the training that were relevant to their roles. All team members received training on research ethics, the importance of confidentiality of participants and data, safety, and guidelines for recruiting. 

 Community partners identified two WPD peer facilitators who would be trained to implement HP groups. In addition to the 10-day face-to-face training series we also provided additional site specific face-to-face and telephone training support as needed as part of peer facilitator training. Training specific to implementation of groups included the following areas: understanding depression and anxiety; crisis intervention; suicide assessment and intervention; violence and abuse assessment and intervention; and responding to trauma. Peer facilitators also learned about the concept of fidelity and reviewed study procedures for facilitator adherence and competence. Methods of training included instructional videos, self-paced readings, and face-to-face training with mock sessions and role plays. Training was provided by persons from the disability community with recognized expertise in specific areas as well as by Psychiatric Mental Health Nurse Practitioners. 

 Each community partner identified two to three data collectors to conduct the screening and enrollments and complete data collection interviews. These team members received training guidelines for how to screen and enroll participants including informed consent procedures. Team members demonstrated proficiency conducting three mock screening and enrollment interviews prior to starting actual data collection. We reviewed and practiced safety protocols in detail, often using role play scenarios. We also reviewed protocols related to maintaining follow-up contact with participants in-between time points. Participants who required additional support were provided with one-on-one tutoring until they mastered all content.

#### 3.3.3. Supervision of Lay Leaders

Peer facilitators received clinical supervision as a group for two hours each week with a highly experienced doctorally prepared Psychiatric Mental Health Nurse Practitioner throughout the duration of the intervention period. This clinical supervisor was also available for additional consults as needed throughout the week. In addition, the PI, also a Psychiatric Mental Health Nurse Practitioner, was available for emergency consults seven days a week. 

#### 3.3.4. Research Fidelity

We developed tools for measuring intervention fidelity for use specific to this project. These tools included (1) a session specific adherence checklist which included a time measurement index for core elements in each session and (2) a facilitator competence measure. We developed session specific adherence checklists by mapping activities from the facilitator manual. To develop the facilitator competence measure we conducted a literature search for the purpose of identifying existing reliable and valid competence measures. Once we had identified measures with the greatest applicability to the HP curriculum we then adapted items from these existing scales to create the Healing Pathways facilitator competence measure. Next, we pilot tested the competence measure using two independent coders who coded three audio recorded practice sessions. To assess the fidelity of actual group sessions we randomly selected groups for coding each week of the 14-week program. Two independent coders listened to audio recordings of these sessions. Coding was subsequently discussed with the PI. In addition, to elicit qualitative data relevant to intervention fidelity and quality, we asked both coders and group facilitators to complete a narrative for each session. While relevant to intervention fidelity, we used this qualitative information primarily as a means of assessing the quality of sessions, thereby providing a feedback loop for quality improvement. Using these methods of measurement we implemented the HP program with a facilitator adherence of 87% and competence of 2.79 (3 point scale, 3 = most, and 1 = least competent).

#### 3.3.5. Recruitment

All study activities and procedures were approved by the University Internal Review Board. We also obtained a Certificate of Confidentiality from the Department of Health and Human Services to provide additional protection to participants. We used detailed protocols to ensure the safety of participants including protocols addressing suicidal ideation and violence and abuse. All collaborating partners recruited participants for the study using a variety of strategies. We used a flier describing the study and its target population of “women with physical disabilities who have depressive symptoms” as part of our recruitment efforts. Thus, participants who responded to the fliers self-selected based on their perception of themselves as women who experienced both physical disability and depression. Our recruitment strategies included a direct mailing via a durable medical equipment mailing list, advertisements on Craig's List, and active community recruiting at places such as grocery stores, bus stops, and local disability interest groups including the Multiple Sclerosis Society and United Cerebral Palsy. 

 In addition to recruiting, each collaborating partner was involved in screening and enrolling participants for the study. We used a two-step informed consent process at screening and enrollment interviews. First we obtained consent for screening and completed screenings. We then obtained consent for enrollment and completed enrollment interviews for those women who were deemed eligible and wanted to participate. Screening and enrollments took place either at the local community partner offices or at a location of the participants' choosing. 

 After recruitment, screening and enrollment processes were completed we randomized participants to the intervention or a wait-list control group. We used SPSS as the method of random assignment. The wait-list control group was smaller than the initial group to minimize the number of women who would have to wait for receipt of mental health services as a result of study participation. Women were notified of their random assignment by the same team member who had conducted their screening interviews either in person or over the phone. 

#### 3.3.6. Data Collection

We used face-to-face interviews to collect data. Instruments were completed by interviewers who had participated in the aforementioned 10-day training. Participants randomized to the initial treatment group participated in data collection interviews for time points 1–4. During this same time period participants randomized to the wait-list control group completed identical pre- and posttests without participating in the intervention. Participants randomized to the wait-list control group then participated in a different version of the HP program and participated in an additional three data collection time points (time points 5–7). Because we tested two different versions of the program, we have chosen not to combine the results. Therefore we report on time points 1–4 here. Accordingly for the purposes of this paper the wait-list control group is hereafter referred to simply as the control group. The four time points reported on here include the pretest (time point 1), a posttest conducted immediately upon completion of the HP program (time point 2), six-week postintervention (time point 3), and three-month postintervention (time point 4). 

## 4. Outcomes and Measures

All outcome variables and covariates were derived primarily from discussions among the HP partners and a review of empiric literature. We selected our measures and the specific subscales used from those measures based on the goals and outcomes of the HP program including the skill-building focus of the four core areas described above. Our primary outcome measure of depressive symptoms, our secondary outcome measures of coping, health behavior, global self-esteem, body and sexual-self-esteem, and loneliness, and our measures of covariates are described below. 

### 4.1. Depressive Symptoms

We measured our primary outcome variable of depressive symptoms using the Center for Epidemiologic Depression Scale (CES-D). The CES-D is a 20-item measure that assesses mood during the past week using a 4-point Likert scale ranging from 0 = rarely or none (less than a day) to 3 = most or all of the time (5 days). Four of the 20 items are reverse scored, and the total measure is scored by summing all items. A score of 16 or greater signals possible depression. The CES-D has demonstrated excellent reliability with Cronbach's alphas ranging from 0.85 in community samples to 0.90 in psychiatric samples [38]. In a previous study we conducted with 165 WPD we used the CES-D and obtained a Cronbach's alpha of 0.92 [39]. 

### 4.2. Coping

We measured coping using selected sub-scales of the Brief COPE. The Brief COPE contains 30 items to assess a broad range of coping and problem-solving responses with 14 two-item sub-scales. The inventory includes some responses that are expected to be dysfunctional, as well as some that are expected to be functional. We selected six of the 14 sub-scales for use based on their relevance to HP program core skill areas. These include substance abuse, emotional support, positive reframing, self-blame, active coping, and instrumental support. The COPE has been used in previous studies with WPD [29, 40] as well as with depressed women [41]. For each item, the respondent is asked to indicate the frequency of each behavior/thought process, ranging from 1 = I usually do not do this at all to 4 = I usually do this a lot. The subscales are scored using the mean of 4 items. Cronbach's alphas for COPE subscales range from 0.63 to 0.80 [41]. 

### 4.3. Health Behavior

We measured health behavior using the Health Promoting Lifestyle Profile II (HPLP-II). The HPLP-II is a 52-item global measure of health behavior. It contains six subscales. We selected three of the six sub-scales for use based on their relevance to HP program core skills areas including spirituality, interpersonal relations, and stress management. For each item, the respondent indicates the frequency of each behavior ranging from *N* for never (=1) to *R* for routinely (=4). Scoring uses the mean of items on each subscale. Cronbach's alphas on HPLP-II subscales range from 0.69 to 0.86 [42, 43]. 

### 4.4. Rosenberg Self-Esteem Scale

We measured global self-esteem using the Rosenberg Self-Esteem Scale (RSES). The RSES is a 10-item measure of one's overall evaluation of the self. The measure uses a 4-point Likert scale ranging from 3 = strongly agree to 0 = strongly disagree. The total measure is scored by summing all items The RSES has been used with WPD in other studies with Cronbach's alphas reported from 0.70 to 0.90 [20].

### 4.5. Body and Sexual Self-Esteem

We measured body and sexual self-esteem using the Physical Disability Sexual and body Esteem Scale (PDBSE). The PDBSE is a 10-item measure of the capacity to feel positive about one's sexuality and body while living with a physical disability [31, 44]. The measure uses a 4-point Likert scale ranging from 1 = strongly agree to 4 = strongly disagree. The total measure is scored by summing all items. In our previous study with 165 WPD, we obtained a Cronbach's alpha of 0.90 [39].

### 4.6. Loneliness

We measured loneliness using the UCLA Loneliness scale-Version 3 (UCLA-LS). The UCLA-LS is a 20-item scale that measures levels of loneliness in everyday life. It includes 9 positive (nonlonely) and 11 negative (lonely) items, randomly distributed throughout the instrument. Participants rate each item on a 4-point Likert scale ranging from 1 = never to 4 = always. The UCLA-LS has previously been used successfully with WPD and women with depression [45]. Nine of the items are worded positively and reversed scored. The total measure is scored by summing all items. Cronbach's alpha for the scale range from 0.89 to 0.94 [46, 47]. 

## 5. Measurement of Covariates

Several studies have identified significant relationships among pain, fatigue, and depressive symptoms in a variety of populations. Specifically, it has been observed that severity of depressive symptoms varies in proportion to the level of pain and/or fatigue experienced [48–52]. Despite the well-established associations between pain, fatigue, and depressive symptoms, it is not clearly understood how these variables interact with physical disability [50, 52]. Substance abuse (as an identified problem rather than as a coping style) has also been shown to be both common in people with disabilities [53] and associated with depression [54, 55]. Because these variables could potentially influence depressive symptoms in WPD, we included them in our analysis as covariates. Other covariates include age group, socioeconomic status (SES), and physical dependency. We used a demographic questionnaire to collect data on age and SES. The remaining measures are described below.

### 5.1. Physical Dependency

We measured physical dependency using the Stanford Health Assessment Questionnaire (HAQ). The HAQ is an 8-item measure of ability [56]. Participants are asked to rate each item on a 4-point Likert scale ranging from 0 = without difficulty to 3 = unable to do. The measure is scored using the mean of 8 items. In our previous study with 165 WPD we obtained a Cronbach's alpha of 0.92 [39]. 

### 5.2. Substance Abuse

We measured substance abuse using the CAGE-AID. The CAGE-AID is a 4-item screening measure that has been used with WPD and depressed women in numerous studies [57]. The CAGE-AID screens for alcohol or drug problems based on the CAGE alcohol screen. Respondents are asked to answer 1 = yes and 0 = no to four questions. The CAGE-AID is scored by counting the number of items answered yes. Score of 1 = possible problem and 2 = probable problem. The CAGE-AID has exhibited strong sensitivity and specificity, ranging from 60 to 95% and from 40 to 95%, respectively [57, 58]. 

### 5.3. Pain and Fatigue

To assess pain and fatigue we used verbal rating scales (VRS) of which have been used extensively in clinical practice and research with WPD [59–63]. Participants were asked “On a 0 to 10 scale, where “0” equals no fatigue and “10” equals the worst fatigue imaginable, how severe has fatigue been, on average, during the past week?” The same approach scale was used to assess pain. The VRS for fatigue's test-retest reliability is strong (0.93). Cronbach's alpha of the VRS for pain is 0.88 [63]. 

### 5.4. Statistical Analysis

We used SPSS 16.0 and SAS 9.2.2 to conduct our statistical analyses. We computed means, standard deviations, and frequencies for numerical and categorical variables. At the baseline, we perform the comparison for the group difference using Chi-square/Fisher's Exact test (for qualitative variables and two independent sample *t*-test/Wilcoxon Rank Sum test (for quantitative variables) when appropriated. To test hypothesis (1)  WPD receiving the intervention will demonstrate a greater improvement in depressive symptom scores compared to participants in the control group, we used a mixed effects model in which the outcome of interest is the depressive symptom score, the two fixed-effects are group treatment effect with two levels (intervention versus control), and the time effect has four levels (baseline, posttest, 6-week followup, and 3-month followup). Subject specificity served as a random effect in this model. An interaction treatment-by-time effect was examined to test if the intervention improved the outcome of depressive symptoms. Similarly, to test hypothesis (2)  WPD receiving the intervention will demonstrate a greater improvement in coping, health behavior, global self-esteem, body image and sexual self-esteem, and loneliness compared to participants in the control group, we used a mixed effects model on these secondary outcomes. Significant group-by-time interactions, such that the intervention group exhibited significant improvement in the secondary outcomes compared to the control group would provide support for hypothesis 2. 

 To address the research question: *What are the relative contributions of changes in coping, health behavior, global self-esteem, body image and sexual self-esteem, and loneliness to changes in depressive symptoms?*  we applied a mixed effects model in which the depressive symptom score is the outcome of interest, and each secondary outcome served as a primary predictor. We also included the group treatment, time, and the interaction treatment-by-time in the model as adjusting factors while examining the association. Those secondary outcomes having a significant association with depressive symptom scores were included in the multivariable model for association testing. If slope testing for each of these secondary outcomes was significant, an association between the change in that particular secondary outcome and change in depressive symptoms was established. We also included age and SES in the model as adjusting covariates/factors. We then compared the standardized estimates (slope coefficient estimates) for each significant slope parameter to determine the relative strength of the secondary outcomes in predicting changes in depressive symptoms. 

 We conducted a per protocol (PP) analysis using mixed-design ANOVAs with complete data, and an intent-to-treat (ITT) analysis using hierarchical linear modeling (HLM). We found ITT provided us with greater power, for example, smaller *P* values. In addition, with ITT, we examined linear and quadratic terms of time effect and found that the quadratic model did not capture the time effect, for example, *P* values >  .05. In fact, we studied the overall (mean) trajectories over time of the outcomes of interest and found that the quadratic curve was not supportive. Taking all these observations into account, to maximize all available data, our analysis addressing aims 1 and 2, and research question 1 is based on a mixed effects model. To our knowledge, this statistical method has been widely used in longitudinal studies for the last several decades. The advantage of this method is its flexibility in dealing with missing values; a missing observation does not cause the deletion of other observations from the same subject in the analysis. Thus, we made use of all available data, capturing greater statistical power for hypothesis testing. 

 Because mixed effects modeling is based on a maximum likelihood approach, the quantity of sum of squares due to each effect or error cannot be obtained. As such it is not possible to compute partial *η* ^2^ as an effect size measure with this approach. Therefore to compute the effect size, we used a general mixed ANOVA model. In sequence, this statistical method was applied to each aim and research question discussed in detail below. The level of significance of an effect was tested at an alpha of 0.05. If the *P* value of a test was between 0.05 and 0.1, we considered that the effect had a trend toward significance. Because we used a mixed effects model in which a missing value of one observation does not cause the deletion of the observed data of the related-subject from the analysis and we had very few missing values, it was not necessary to use an imputation technique. 

## 6. Results

Our group retention rate was 81% overall, more specifically 95% in the intervention group; 67% in the control group. We examined the distributions of age for each group, and the assumption of normality was reasonably met. The means and standard deviations were 51.59 ± 10.18 for the intervention and 52.09 ± 10.90 for the control group. We performed a two independent sample *t*-test as the comparison between the two groups; there was no significant difference in age between the intervention and control groups (*P* = 0.835). Participants' age ranges were 28–80 and 19–71 in the intervention and control groups, respectively. We used income as a proxy for SES. To compare the distributions in SES between groups, we subgrouped the number of categories to 3; specifically, we kept the first two categories $0–$10,000 and $10,000–$20,000 and created a new category for those whose salary was greater than $20,000. We performed a Chi-square test as the comparison between the two groups; there was no significant difference in SES between the intervention and control groups (*P* = 0.338). With regard to ethnicity, 75% (33/44) versus 77.14% (27/35) of the intervention versus the control group, respectively, were European-American; there was no significant difference between the two groups (*P* = 0.82). We also conducted further comparisons between the two groups on the other characteristics/covariates, that is, education (*P* = 0.77); employment (*P* = 0.06); marital status (*P* = 0.07); disability severity (*P* = 0.15); and receiving assistance from others (*P* = 0.28). Finally, 71% and 56% of participants in the intervention and control groups, respectively, reported arthritis or musculoskeletal disorders (*P* = 0.18), 24% and 35% reported neurological disorders (*P* = 0.29), and 45% and 40% reported other conditions as their disability types (*P* = 0.58). In summary, there was no statistical significant difference in demographic and disability characteristics between the intervention and control groups. See Table 1 for more details.

 We checked the range of values for the other variables and found them to be in the reasonable range. Since the data of our primary outcome (depressive symptoms), secondary outcomes (coping sub-scales (substance abuse, emotional support, positive reframing, self-blame, active coping, instrumental support), health behavior sub-scales (spirituality, interpersonal relations and stress management), global self-esteem, body image and sexual-self-esteem, and loneliness), and other covariates (physical dependency, substance abuse problem, and pain and fatigue) were collected over time, the distributions of each measure at each time point were studied and are summarized in Tables 2(a) and 2(b). We also performed a comparison between the two groups on the primary outcome, depressive symptoms, at baseline and found a significant difference (*P* = 0.04). As shown in Tables 2(a) and 2(b), the (mean ± sd) of depressive score of the two groups at the pretest are 1.71 ± 0.52 versus 1.37 ± 0.5 for the intervention versus control group, respectively. These mean scores indicate, on average, that the intervention group had more severe depression (higher mean score) at baseline. However, the situation was reversed at the final post-test; that is, the depressive score was lower in the intervention group (1.08 ± 0.64) compared to the control group (1.38 ± 0.5). This observation was confirmed with our testing for the interaction between time and group treatment on the depression measure outcome (see below).

**Figure 1:**
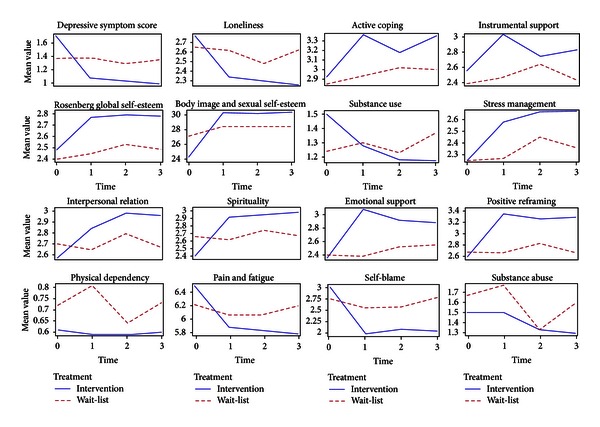


 Examination of the predicted mean curves over time between the intervention and control groups suggests that interaction effects occurred in depressive symptom score, loneliness, body image and sexual self-esteem, substance use, stress management, interpersonal relations, spirituality, emotional support, positive reframing, and self-blame. We did not identify interaction effects in active coping, instrumental support and global self-esteem (see Figure 1). These suggestions were confirmed by testing for interaction terms between time and group treatment as shown in Table 3. Furthermore, in Figure 1, in the intervention group, we saw a sharp increase/decrease from the pre-test (Time 0) to post-test (Time 1) and that this pattern was either continued or stabilized at three-month followup (Time 4). This kind of pattern occurred in most scales that showed the existence of the aforementioned interaction. On the other hand, in the control group, most mean curves were either flat or unstable over time. Please see Figure 1 for information on the mean curves on covariates, for example, physical dependency, substance abuse, and pain and fatigue.

 From Table 3, we found that there was a significant interaction effect of treatment by time on depression symptoms scores (*P* < 0.0001), indicating the efficacy of the intervention program. Furthermore, the plot for depression symptoms scores in Figure 1 shows a sharp decrease from the pretest to posttest and that is stabilized at three-month followup in the intervention group. On the other hand, the mean curve is flat over time in the control group. For coping skills,we found a series of significant treatment-by-time interaction effects: substance use (*P* = 0.028); emotional support (*P* = 0.005); positive reframing (*P* = 0.002); andself-blame (*P* = 0.0004). We did not find enough evidence to support hypothesis 2 on active coping (*P* = 0.325) and instrumental support (*P* = 0.144), respectively. For health behavior, we found significant interaction treatment-by-time effects on all three selected sub-scales as follows: spirituality (*P* < 0.0001); interpersonal relations (*P* = 0.0002); and stress management (*P* = 0.028). We also found strong support for hypothesis 2 on loneliness when testing the interaction treatment-by-time effect (*P* < 0.0001). Testing of the interaction treatment-by-time effect for body image and sexual self-esteem also indicated a significant interaction (*P* = 0.013). However, testing of the interaction treatment-by-time effect for global self-esteem (*P* = 0.1292) indicated that the interaction effect was insignificant (see Table 3).

 Regarding the effect size difference between the groups, overall, depressive symptom score, loneliness, and spirituality were strong, ranging from 0.1 to 0.16; interpersonal relations, self-blame, positive reframing and emotional support were fairly strong, ranging from 0.06 to 0.09; substance use, stress management, and body image and sexual self-esteem were moderate, ranging from 0.04 to 0.05; and active coping, instrumental support, and global self-esteem were fairly weak, ranging from 0.01 to 0.025 (see Table 3).

 We also investigated the covariates—physical dependency, substance abuse problem, and pain and fatigue. We found that substance abuse and the fatigue scales were significant, and thus we included them in the multivariable model for association analysis (see below). 

 When examining the associated effects of each secondary outcome on the depression symptom score, the slope testing results showed that five of six selected coping sub-scales were significantly associated with changes in depressive symptom scores: substance use [*F*(1,208) = 4.31, *P* = 0.039, partial *η* ^2^ = 0.065]; emotional support [*F*(1,209) = 31.65, *P* < 0.0001, partial *η* ^2^ = 0.089]; instrumental support [*F*(1,209) = 7.69, *P* = 0.006, partial *η* ^2^ = 0.024]; positive reframing [*F*(1,209) = 20.42, *P* < 0.0001, partial *η* ^2^ = 0.008]; and self-blame [*F*(1,209) = 58.92, *P* < 0.0001, partial *η* ^2^ = 0.104]. Active coping was insignificantly associated with changes in depressive scores [*F*(1,208) = 1.92, *P* = 0.168, partial *η* ^2^ = 0.002]. We also found that all three of the selected sub-scales in health behavior were significantly associated with the changes in depressive symptom scores: spirituality [*F*(1,209) = 61.23, *P* < 0.0001, partial *η* ^2^ = 0.159]; interpersonal relations [*F*(1,209) = 18.05, *P* < 0.0001, partial *η* ^2^ = 0.058]; and stress management [*F*(1,209) = 38.77, *P* < 0.001, partial *η* ^2^ = 0.093]. Furthermore, loneliness was strongly associated with the changes in depressive symptom scores, its slope testing has [*F*(1,209) = 84.32, *P* < 0.0001, partial *η* ^2^ = 0.215]; as has body image and sexual self-esteem [*F*(1,209) = 9.41, *P* = 0.002, partial *η* ^2^ = 0.094]. Global self-esteem was barely significantly associated with the changes in depressive scores, and its slope testing has [*F*(1,209) = 3.84, *P* = 0.05, partial *η* ^2^ = 0.0026].

 The results of the multivariable model which includes eleven secondary outcomes that are singly significantly associated with the primary outcome of depressive symptoms indicate that seven variables remained significant model. We found loneliness to be the strongest in contributing to changes in the outcome of depressive symptoms with a positive association, its standardized estimate of 0.3827. This means that for every 1 point increase in loneliness, we would expect to see a 0.38 increase in the depressive symptom score. Interpersonal relations came in as the second strongest with a positive association, its standardized estimate of 0.3377. With this unexpected finding we found that for every 1 point score increase in interpersonal relations there was roughly a 0.34 score increase in the depression symptom score. The third largest contributor was spirituality with a negative association, its standardized estimate of −0.2799. For every 1 point increase in spirituality, we would expect to see a 0.28 score decrease in the depression score. Emotional support and self-blame came in fourth and fifth, with their negative/positive association, the standardized estimates of −0.135 and 0.144, respectively. Their interpretations were opposite; for every 1 point increase in emotional support, we would expect roughly a 0.14 score decrease in the depression symptom score, and for every 1 point increase in self-blame we would expect roughly a 0.14 score increase in the depression symptom score. Sixth was positive reframing with a negative association, its standardized estimate of −0.077. Every 1 point increase in positive reframing contributed roughly a 0.077 decrease to the depression score. Seventh was substance use with a fairly weak association, its standardized estimate of 0.07. Every 1 point increase in substance use contributed roughly a 0.07 score increase to the depression score. 

### 6.1. Postprogram Focus Group Feedback

 We conducted focus groups at the end of the 14-week program with each therapy group. We asked participants to evaluate the program in terms of content, process, and outcomes. With regard to content all of the programmatic content was viewed as important; however, it was suggested that content related to sexuality be made optional. Process feedback strongly suggested that women in the groups wanted more time for personal sharing during each session and that overall there was a belief that a 14-week program was too short to adequately cover all important topics. Finally, we received overwhelmingly strong and positive feedback about the quality of the program and the transformative impact it had had on women's lives. Women made many positive changes during the program including leaving abusive relationships, expanding their social networks, and achieving gainful employment. 

## 7. Discussion

 Depression in WPD is a common problem; that is, associated with significant morbidity and mortality. Although several nonpharmacologic treatments for depression are available, we are unaware of any that specifically address the psychosocial issues faced by WPD cross-disability. The HP program offers a novel approach to addressing depression in WPD as a cooccurring condition that draws on both mental health theories and peer empowerment approaches to treatment. 

 Despite randomization we found differences at baseline on many variables between the intervention and control groups including a statistically significant difference in depressive symptoms with the treatment group having higher depressive symptom scores. This is a limitation of our study. Despite this limitation our findings provide initial support for the HP program's efficacy in treating depressive symptoms as well as improvements in selected secondary outcomes of coping (substance use, emotional support, positive reframing, self-blame), health behavior (spirituality, stress management), self-esteem (body and sexual), and loneliness, all important for WPD. In addition, the pattern of results suggests that HP's four core skill areas may explain these outcomes. Identifying and addressing thoughts and beliefs and identifying and managing emotions (the first two skill areas) are foundational skills to improving interpersonal skills and developing and strengthening coping and problem-solving skills (the last two skill areas). It is these latter two skill areas that are manifested most clearly in the associations between our coping, health behavior, body and sexual self-esteem, and loneliness variables and our outcome variable of depressive symptoms. Interpersonal skills can be said to correspond to the variables emotional support and loneliness while coping and problem-solving can be said to correspond to the variables substance abuse, positive reframing, self-blame, spirituality, and stress management. 

 Three variables were insignificant including active and instrumental coping and global self-esteem. Active coping was not significantly associated with an improvement in depressive symptom scores suggesting that it may lack relevance to the primary outcome of depressive symptoms. Similarly, instrumental coping although singly significantly associated with an improvement in depressive symptoms scores proved insignificant in the multivariate model suggesting it may also lack relevance to the primary outcome when other associated effects are considered jointly. Another possible consideration is the impact of the use of acceptance as a coping strategy. Examination of active and instrumental coping items points to active change efforts, forgoing the possibility of acceptance as a coping strategy (i.e., “doing something about the situation” (active coping) and “getting help and advice from other people” (Instrumental coping). It is possible that acceptance, which was taught as a part of the managing emotions portion of HP, was used by participants as a balancing strategy along with more active strategies resulting in a dilution of the strength of active and instrumental coping approaches. With regard to global self-esteem, improvement in this secondary outcome in the intervention group coupled with the lack of significance suggests that this program element needs strengthening. Focus group participant feedback suggested that a longer program allowing for more in-depth exploration of content would be stronger. This may be an example of program content that was not covered in sufficient depth. 

 In response to the troubling finding that the Interpersonal Relations sub-scale of the Health Promoting Lifestyle Profile II was both significantly improved and associated with an increase in depressive symptom scores we examined the items and the pattern of responses of this scale. In doing so we found that participants in the intervention group strongly improved relative to the control group on every item. Although some of the items seem to be unarguably positive (e.g., “maintaining meaningful and fulfilling relationships with others), others were not necessarily so. For example, “discuss my problems and concerns with people close to me” may be positive in some instances but not in others. Discussing problems with others repeatedly without movement or change could be a sign of dependency. Similarly, discussing problems with others who are close to you but not very supportive could prove detrimental. Therefore some of the items on the interpersonal relations sub-scale, although in theory measuring positive health behaviors, may not be always positive. This may explain at least in part why the improvement in interpersonal relations was associated with an increase in depressive symptoms.

In addition to statistically significant results we also observed significant changes in women's lives during our study. Following study completion, participants in one rural town subsequently went on to establish a mental health drop-in center. Others started their own HP groups. Although we did not formally measure changes in employment, anecdotally, we also observed that many women who were experiencing challenges staying employed reported positive changes in sustaining employment. One woman started her own business. Others sought gainful and meaningful employment. Word of this effect eventually led to establishment of a contract with Oregon Vocational Rehabilitation for services to unemployed WPD with cooccurring depression. We developed an untested employment version of the HP program for this purpose. Depression and employment outcomes of the first year of that program look promising. Of the 14 WPD who completed exit interviews a mean 15.6 point drop in CES-D scores was observed. In addition, of those 14 chronically unemployed women, 8 obtained employment suggesting that testing of the employment version of the HP program may be appropriate at some point in the future. In addition to this employment version, the original HP program (described in this paper) continues to be offered to WPD with cooccurring depression in two Oregon communities. 

 In addition to our work with the original HP program, we are currently engaged in work developing a HP peer facilitator training program to standardize the preparation of peers trained to implement the program using CBPR principles. It is our hope to disseminate the HP program to expand treatment options for WPD with cooccurring depression and in particular to increase options for treatment within disability communities. This approach is consistent with the RE-AIM framework, a model intended to enhance the quality, speed, and public health impact of efforts to translate research into practice in five steps including *reaching* your intended target population, demonstrating *efficacy*, *adoption* by target settings or institutions, *implementation* (i.e., consistency of delivery of intervention), and *maintenance* of intervention effects in individuals and settings over time [64]. We are currently focused on the adoption and implementation components of the model. 

 In conclusion, our study provides support for the efficacy of the HP program in addressing depressive symptoms in WPD and some selected secondary outcomes. Because it is specifically designed for WPD, the HP program has potential to provide WPD with an alternative treatment option for depressive symptoms, that is, culturally relevant and accessible. We hope that disability service organizations, WPD community groups, and rehabilitation counselors find this freely available program useful to their work. 

## Figures and Tables

**Table 1:** 

Demographic Characteristics				
Categorical variables	Intervention	Control	All	*P* value
	*N* (%)	*N* (%)	*N* (%)	

Ethnicity				
African American/black	2 (4.55%)	0 (0%)	2 (2.53%)	0.82*
Unknown	0 (0%)	3 (8.57%)	3 (3.8%)	
Asian	0 (0%)	1 (2.86%)	1 (1.27%)	
Hispanic	0 (0%)	2 (5.71%)	2 (2.53%)	
Multiracial	7 (15.91%)	2 (5.71%)	9 (11.39%)	
White	33 (75%)	27 (77.14%)	60 (75.95%)	
Native American	2 (4.55%)	0 (0%)	2 (2.53%)	
Employment				
(1) No	36 (81.82%)	22 (62.86%)	58 (73.42%)	0.058
(2) Yes	8 (18.18%)	13 (37.14%)	21 (26.58%)	
Marital status				
Single	34 (79.07%)	21 (60%)	55 (70.51%)	0.0662
Married, living together	9 (20.93%)	14 (40%)	23 (29.49%)	
Income				
$0 to $30,000	36 (81.82%)	28 (80%)	64 (81.01%)	0.338*
$31,000 to $50,000	5 (11.36%)	3 (8.57%)	8 (10.13%)	
$51,000 to $70,000	2 (4.55%)	3 (8.57%)	5 (6.33%)	
$71,000 to $101,000	1 (2.27%)	1 (2.86%)	2 (2.53%)	
Disability severity				
1	1 (2.27%)	1 (2.86%)	2 (2.53%)	0.15
2	14 (31.82%)	18 (51.43%)	32 (40.51%)	
3	29 (65.91%)	16 (45.71%)	45 (56.96%)	
Receive assistance from others				
(1) No	6 (13.64%)	2 (5.56%)	8 (10%)	0.28
(2) Yes	38 (86.36%)	34 (94.44%)	72 (90%)	

Continuous variables	Mean ± SD	Mean ± SD	Mean ± SD	

Age	51.59 ± 10.18	52.09 ± 10.9	51.81 ± 10.44	0.82
Education	14.02 ± 2.24	14.17 ± 2.32	14.09 ± 2.26	0.77

Primary disability	Intervention	Wait-list	All	
	*N* (%)	*N* (%)	*N* (%)	

Arthritis, other musculoskeletal disorders (yes)	32 (71.11%)	21 (56.76%)	53 (64.63%)	0.18
Neurological disorder (yes)	11 (24.44%)	13 (35.14%)	24 (29.27%)	0.29
Others (yes)	21 (46.67%)	15 (40.54%)	36 (43.9%)	0.58

*is performed on the regrouped categories. See the text for more details.

**Table:** (a)

	Pretest			Posttest			6-week followup			3-month followup		
Measures	Time 0			Time 1			Time 2			Time 3		
	*N*	Mean	SD	*N*	Mean	SD	*N*	Mean	SD	*N*	Mean	SD
Primary outcome												
Depressive symptom score	44	1.71	0.52	40	1.08	0.64	42	1.03	0.61	41	0.99	0.54
Secondary outcome												
(i) Coping and problem-solving												
(a) Active coping	44	2.92	0.86	39	3.36	0.69	42	3.18	0.75	41	3.35	0.64
(b) Substance use	44	1.5	0.93	39	1.28	0.65	42	1.18	0.43	41	1.17	0.4
(c) Emotional support	44	2.35	0.8	40	3.08	0.79	42	2.92	0.87	41	2.88	0.88
(d) Instrumental support	44	2.55	0.87	40	3.03	0.84	42	2.75	0.86	41	2.83	0.86
(e) Positive reframing	44	2.59	0.88	40	3.35	0.77	42	3.26	0.77	41	3.29	0.8
(f) Self-blame	44	3.02	0.81	40	1.99	0.65	42	2.08	0.73	41	2.05	0.84
(ii) Health behavior												
(a) Spirituality	44	2.4	0.51	40	2.92	0.5	42	2.94	0.54	41	2.98	0.55
(b) Interpersonal relation	44	2.57	0.47	40	2.84	0.46	42	2.98	0.48	41	2.96	0.5
(c) Stress management	44	2.25	0.54	40	2.58	0.45	42	2.67	0.49	41	2.67	0.56
(iii) Loneliness	44	2.77	0.46	40	2.34	0.5	42	2.3	0.48	41	2.26	0.48
(iv) Rosenberg global self-esteem	44	2.48	0.55	40	2.77	0.49	42	2.79	0.5	41	2.78	0.53
(v) Body Image and sexual self-esteem	44	24.25	7.46	40	30.3	7.4	42	30.21	8.14	41	30.39	9.09
Covariates												
(i) Physical dependency	44	0.61	0.34	40	0.59	0.37	42	0.59	0.39	41	0.6	0.37
(ii) Substance abuse	44	1.5	1.45	40	1.5	1.48	42	1.33	1.37	41	1.29	1.45
(iii) Pain and fatigue	44	6.5	2.16	40	5.88	2.43	42	5.83	2.29	41	5.78	2.21

**Table:** (b)

Measures		Pretest		Control/nonintervention									Intervention								
				Posttest			6-week followup			3-month followup			Posttest			6-week followup			3-month followup		
		Time 0		Time 1			Time 2			Time 3			Time 4			Time 5			Time 6		
	*N*	Mean	SD	*N*	Mean	SD	*N*	Mean	SD	*N*	Mean	SD	*N*	Mean	SD	*N*	Mean	SD	*N*	Mean	SD
Primary outcome																					
Depressive symptom score	36	1.37	0.5	33	1.38	0.5	32	1.29	0.7	30	1.35	0.56	20	1.01	0.6	26	1.19	0.73	24	1.08	0.6
Secondary outcome																					
(i) Coping and problem-solving																					
(a) Active coping	36	2.85	0.8	33	2.94	0.9	32	3.02	0.96	30	3	0.81	20	3.48	0.6	26	3.29	0.81	24	3.23	0.85
(b) Substance use	36	1.24	0.6	33	1.3	0.8	32	1.23	0.75	30	1.37	0.81	20	1.08	0.2	26	1.17	0.62	24	1.06	0.22
(c) Emotional support	36	2.4	0.8	33	2.38	1	32	2.52	0.91	30	2.55	0.86	20	3.15	0.8	26	2.88	0.98	24	2.9	0.83
(d) Instrumental support	36	2.38	0.9	33	2.47	1	32	2.64	0.99	30	2.43	0.98	20	3.15	0.8	26	2.67	0.99	24	2.69	0.83
(e) Positive reframing	36	2.68	0.9	33	2.67	1	32	2.83	0.89	30	2.67	0.79	20	3.23	0.7	26	2.73	0.96	24	2.79	0.88
(f) Self-blame	36	2.76	1.1	33	2.56	1.1	32	2.58	1.06	30	2.78	1.1	20	2.08	0.7	26	2.15	0.89	24	2.27	1.01
(ii) Health behavior																					
(a) Spirituality	36	2.66	0.6	33	2.62	0.6	32	2.74	0.58	30	2.67	0.6	20	3.08	0.5	26	3.02	0.54	24	2.95	0.55
(b) Interpersonal relation	36	2.7	0.5	33	2.65	0.5	32	2.79	0.56	30	2.67	0.47	20	2.97	0.4	26	2.97	0.54	24	2.89	0.55
(c) Stress management	36	2.25	0.4	33	2.27	0.6	32	2.45	0.54	30	2.36	0.56	20	2.69	0.5	26	2.53	0.61	24	2.51	0.59
(iii) Loneliness	36	2.65	0.5	33	2.62	0.5	32	2.48	0.44	30	2.62	0.44	20	2.29	0.5	26	2.36	0.45	24	2.45	0.56
(iv) Rosenberg global self-esteem	36	2.4	0.6	33	2.45	0.7	32	2.53	0.65	30	2.49	0.71	20	2.84	0.6	26	2.74	0.75	24	2.72	0.69
(v) Body Image and sexual self-esteem	36	27.11	7.7	33	28.39	8.9	31	28.48	9.34	30	28.43	9.51	20	31.9	7.3	26	31.9	7.68	24	30.27	7.31
Covariates																					
(i) Physical dependency	36	0.72	0.6	33	0.81	0.5	32	0.64	0.48	30	0.73	0.56	20	0.68	0.5	26	0.67	0.54	24	0.66	0.57
(ii) Substance abuse	33	1.67	1.5	31	1.77	1.4	30	1.33	1.45	29	1.59	1.57	20	1.05	1.4	25	1.48	1.64	23	1.39	1.5
(iii) Pain and fatigue	36	6.22	2.1	33	6.06	2.3	32	6.06	1.54	30	6.2	2.27	20	5.55	2.2	26	5.5	1.84	24	4.79	2.04

**Table 3:** Statistical inference on the interaction effect between time and treatment. The analyses are based on the data for the first four time points. That is, intervention group completed the study, and wait-list group completed the control period time of the study.

Measures	*F*-statistic	*P* value	Partial η^2^
Primary outcome			
Depressive symptom score	9.51	<0.0001	0.101
Secondary outcomes			
(i) Coping and problem-solving			
(a) Active coping	1.16	0.3246	0.017
(b) Substance use	3.11	0.0275	0.046
(c) Emotional support	4.37	0.0052	0.056
(d) Instrumental support	1.82	0.1444	0.025
(e) Positive reframing	5.00	0.0023	0.060
(f) Self-blame	6.28	0.0004	0.065
(ii) Health behavior			
(a) Spirituality	13.60	<0.0001	0.155
(b) Interpersonal relation	6.84	0.0002	0.086
(c) Stress management	3.11	0.0275	0.036
(iii) Loneliness	9.44	<0.0001	0.11
(iv) Rosenberg self esteem	1.91	0.1292	0.0248
(v) Body image and sexual self-esteem	3.70	0.0127	0.042
Covariates			
(i) Physical dependency	2.44	0.0655	0.034
(ii) Substance abuse	0.39	0.7610	0.006
(iii) Pain and fatigue	2.75	0.0436	0.040
